# Evidence that HA‐G228S and PB2‐D153V mutations upon viral growth of H3N8 influenza virus are associated with severe pathogenesis in human infections

**DOI:** 10.1111/irv.13169

**Published:** 2023-06-28

**Authors:** Xue Li, Dongliang Cheng, Yueli Dong, Changsong Shi, Bingqian Qu

**Affiliations:** ^1^ Department of Internal Medicine III University Hospital Heidelberg Heidelberg Germany; ^2^ Department of Pediatric, Henan Provincial People's Hospital Zhengzhou University People's Hospital Zhengzhou Henan China; ^3^ Department of Veterinary Medicine Paul Ehrlich Institute Langen Germany; ^4^ European Virus Bioinformatics Center (EVBC) Jena Germany

Dear Editor,

H3N8, a subtype of the Influenza A virus, was first identified in 1963. It has been detected in birds and mammals, such as camels, cats, dogs, horses, pigs and seals.^1^ Reassortment between H3N8 and other H3 viruses in birds and canines as well as human H3N2 viruses, may increase the fitness of an H3N8 virus to humans.^2^, ^3^, ^4^ The hemagglutinin (HA) protein of some avian‐origin H3N8 virus can bind to both avian (α2,3‐) and human receptors (α2,6‐linked sialic acid).^5^

Recently, two human H3N8 infections have occurred in Henan and Hunan provinces in China.^6^, ^7^ The locations are more than 600 km apart, making the two cases epidemiologically unrelated. Both patients were 4‐ and 5‐year‐old boys without underlying diseases, but their symptoms differed. Case 1 developed severe acute respiratory distress syndrome shortly after infection, and he was ventilated for 3 weeks with the assistance of extracorporeal membrane oxygenation. He was also treated with antibiotics, interferonα‐1 and oseltamivir. He recovered after 2 months of intensive care. In contrast, Case 2 had a high fever but recovered after a four‐day hospitalization with oseltamivir and supportive treatment (Figure 1A).

**FIGURE 1:**
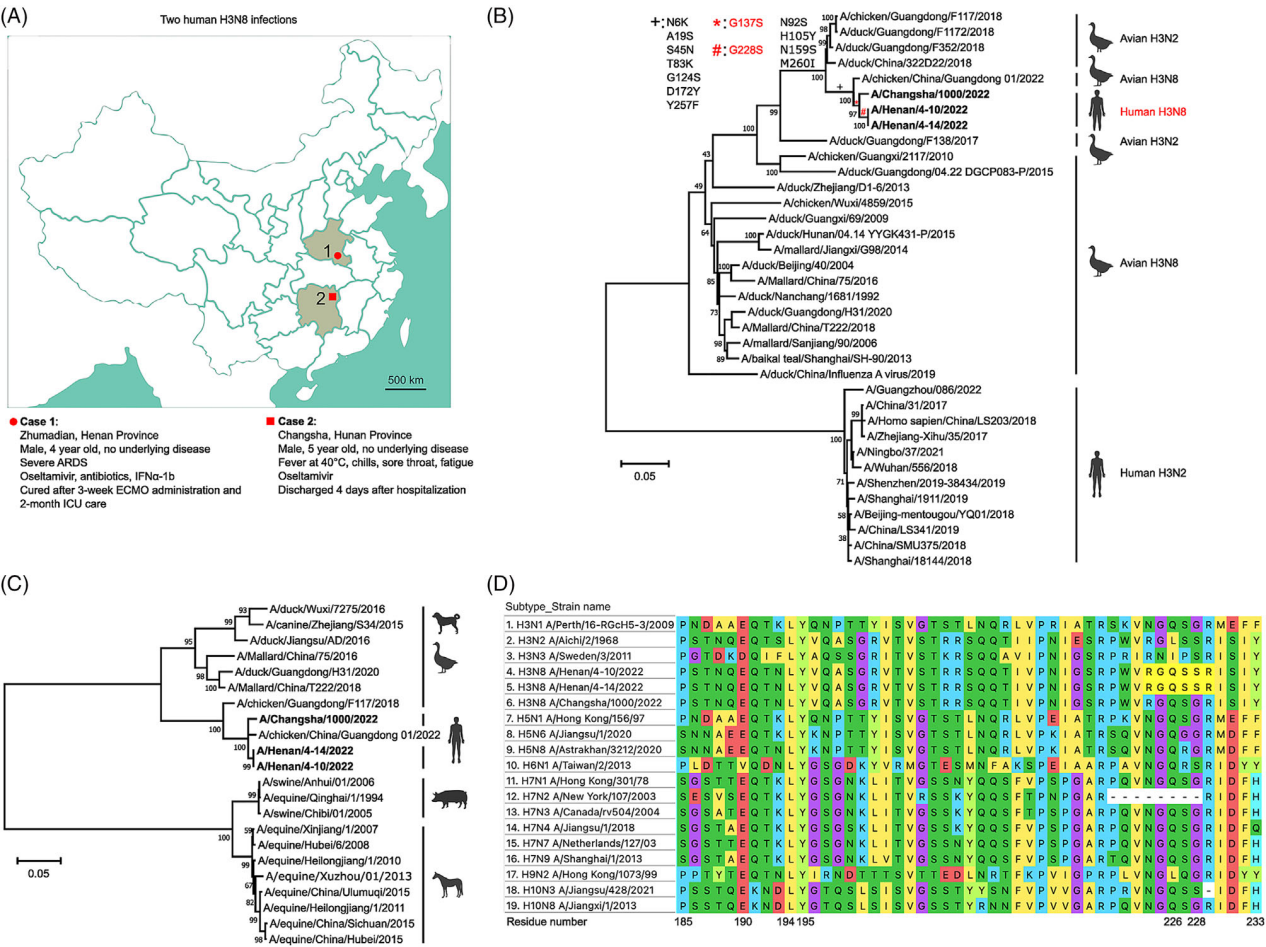
Hemagglutinin (HA) of the first H3N8 human virus shows the highest homology to an avian H3N8 virus and carries a G228S substitution capable of binding to α2,6‐sialic acid receptor. (A) Information on two human H3N8 infections. The legend shows the location, gender, age, symptoms and treatments of both patients. ARDS: acute respiratory distress syndrome. ECMO: extracorporeal membrane oxygenation. ICU: intensive care unit. (B) Phylogenetic analysis of HA genes of human and avian H3N8 and H3N2 viruses. Genetic substitutions of viruses on the branches are depicted. (C) Phylogenetic analysis of human, avian, canine, swine, and equine H3N8 viruses in China. Bootstrap values are shown on the nodes of each branch. Scale bar: the number of nucleotide substitutions per site. (D) Alignment of the HA protein of 17 subtypes that cause human infections. Residues 185‐233 are shown (H3 numbering).

Alveolar lavage fluid and peripheral blood specimens were collected from Case 1 on 10 (*A/Henan/4‐10/2022*) and 14 April of 2022 (*A/Henan/4‐14/2022*) and were analysed using metagenomic next‐generation sequencing (mNGS) through the IDseq Ultra platform.^8^ On a molecular basis, the HA gene of *A/Henan/4‐10/2022* was most closely related to the second human virus (*A/Changsha/1000/2022*) and a chicken H3N8 virus isolated in Hong Kong, with an identity of 98.6%–98.8%. The neuraminidase (NA) gene of *A/Henan/4‐10/2022* was related to avian and human H3N8 viruses found in East Asia and Alaska (Table S1; Figure S1). We further compared the HA sequences of both human cases with those of avian H3N8 and human/avian H3N2 viruses. Phylogenetic analysis showed that both HAs are not homologous with human H3N2 and most avian H3N8 viruses. They showed the highest homology to one avian H3N8 virus and remained close to avian H3N2 viruses (Figure 1B). Furthermore, both HAs of human viruses were related to avian and canine H3N8 viruses, but not ancestral swine and equine viruses (Figure 1C). These data suggest that the virus may have been adapted in birds and canines prior to human transmission.^4^

mNGS analysis revealed a mixed nucleotide at position 759 (R = guanine/adenine) in the HA gene of Case 1, which generates two distinct codons at the amino acid position 228, GGU for glycine (G) or AGU for serine (S). This G228S substitution exists only in Case 1 with severe pathogenesis. Notably, there was an increased proportion of HA‐G228S genomes from 37% (*A/Henan/4‐10/2022*) to 64% (*A/Henan/4‐14/2022*) upon viral replication in vivo. However, it remains unclear whether the original mutation occurred prior to infection or during replication in the child. In both human cases, the glutamine position 226 in the HA proteins remained unchanged. This finding indicates that the HA‐G228S substitution correlated with severe pathogenesis in the first human case.

Q226L/G228S mutations in the HA protein are critical for viral entry into human epithelial cells.^9^, ^10^ In addition, other residues (Y98, H183, E190, L194, Y195 and G225) play a role in receptor affinity.^11^ To evaluate these residues, we aligned HA proteins of 17 subtypes leading to the earliest documented human infections, respectively, and identified four combinations of the two residues: (i) Q226/G228, which retains avian receptor affinity in 11 subtypes, including the second human H3N8 virus; (ii) L(I)226/S228 in human H3N2 and H3N3 viruses (*A/Sweden/3/2011*); (iii) L226/G228 only present in the human H9N2 virus and (iv) Q226/S228 in the first human H3N8 virus as well as H6N1 and H10N3 viruses (Figure 1D). The E190, L194, Y195 and G225 residues are conserved among almost all subtypes. Remarkably, the first human H10N3 infection also caused severe pneumonia in a 41‐year‐old man,^12^ while the first human H6N1 infection caused persistent high fever and shortness of breath in the patient. A chest radiograph taken on day 4 of onset showed an increased bilateral infiltrate in the lower lung. Oseltamivir was immediately given 2 days before influenza infection was confirmed in culture.^13^ Early antiviral treatment may explain why severe pulmonary damage was prevented.

Furthermore, all residues across the viral genome in both cases were compared. The PB2‐E627K substitution is a key determinant of host range and has been critical in the host adaptation of the human H7N9 virus.^14^, ^15^ The mutation present in the severe case (PB2‐627K) but not in Case 2 (627V) could result in more robust replication probably due to enhanced polymerase activity (Table S2). Owing to an E218stop mutation in the NS1 frame, the Case 2 virus has a truncated protein lacking the C‐terminal 20 amino acids compared with the Case 1 virus. The C‐terminus of NS1 protein interacts with several host proteins, leading to an increase in pathogenicity.^16^ It is worth noting that the Case 1 virus only developed two mutations across the whole genome upon viral replication: HA‐G228S as shown above and PB2‐D153V, a mixed nucleotide at position 485 (W = adenine/uracil) that generates two codons at the amino acid position 153, GAT for aspartic acid (D) and GUT for valine (V). However, the potential role of this PB2 substitution in viral pathogenicity remains unknown.

Taken together, compared with the other human H3N8 virus, the virus in the severe case has developed mutations e.g. in the HA, PB2 and NS1 proteins, Upon viral replication in the patient, a larger proportion of HA‐G228S and the genesis of PB2‐D153V correlated with severe pathogenesis.

## AUTHOR CONTRIBUTIONS

**Xue Li:** Data curation; formal analysis; writing—original draft. **Dongliang Cheng:** Data curation; formal analysis. **Yueli Dong:** Methodology; software. **Changsong Shi:** Conceptualization; supervision; writing—review and editing. **Bingqian Qu:** Conceptualization; supervision; writing—review and editing.

## FUNDING INFORMATION

This study received no specific grant from any funding agency. The authors received no financial support for the study, authorship, and/or publication of this article.

## CONFLICT OF INTEREST STATEMENT

All authors declare that they have no conflicts of interest.

### PEER REVIEW

The peer review history for this article is available at https://www.webofscience.com/api/gateway/wos/peer-review/10.1111/irv.13169.

## Supporting information


**Figure S1.** Neuraminidase (NA) genes of the H3N8 human viruses are closely related to H3N8 avian viruses but not H3N2 viruses in China. Phylogenetic tree analysis of NA segments of the H3N8 human viruses (top 3), 13 H3N8 avian viruses (on the top branch), one H3N2 human virus (LS203) and 13 H3N2 avian viruses (on the bottom branch). Scale bar shows the amount of substitutions in the nucleotides.

**Table S1.** Nucleotide sequences of viruses closely related to A/Henan/4‐10/2022(H3N8) in the GenBank and GISAID databases.

**Table S2.** Molecular comparison of all viral residues of the two human H3N8 viruses.

## Data Availability

The data that support the findings of this study are available from the corresponding author upon reasonable request.
