# Supplementary figures and images for: Evidence that HA‐G228S and PB2‐D153V mutations upon viral growth of H3N8 influenza virus are associated with severe pathogenesis in human infections
Source: Influenza Other Respir Viruses. 2023 Jun 28;17(6):e13169. doi: 10.1111/irv.13169 (PMC10303683; doi:10.1111/irv.13169)

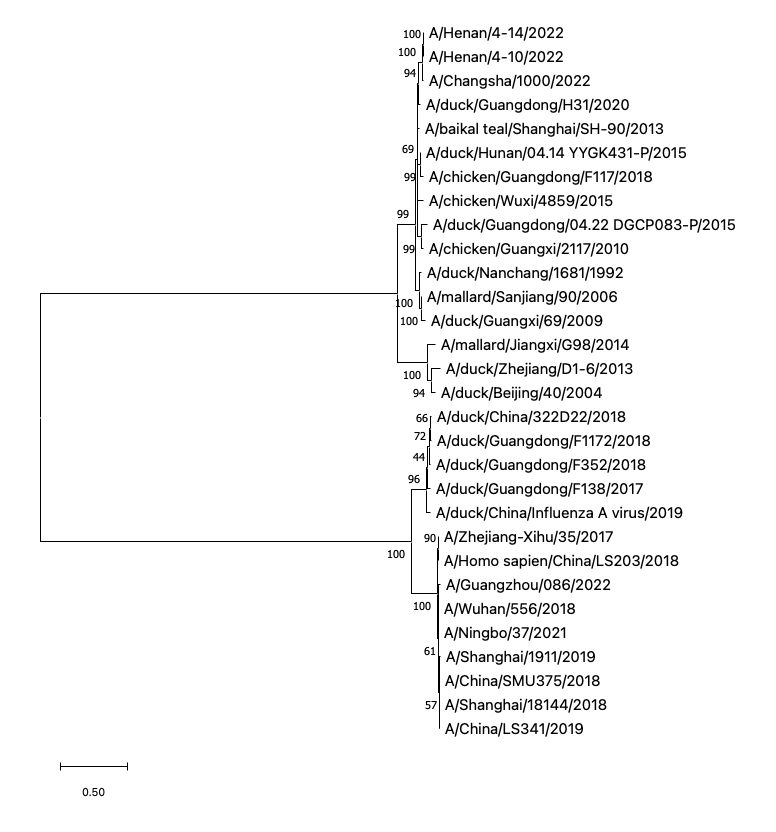

Supplement: Supplementary file 1 — Figure S1. Neuraminidase (NA) genes of the H3N8 human viruses are closely related to H3N8 avian viruses but not H3N2 viruses in China. Phylogenetic tree analysis of NA segments of the H3N8 human viruses (top 3), 13 H3N8 avian viruses (on the top branch), one H3N2 human virus (LS203) and 13 H3N2 avian viruses (on the bottom branch). Scale bar shows the amount of substitutions in the nucleotides. [file IRV-17-e13169-s001.tif]
