# Supplementary material for: Evidence that HA‐G228S and PB2‐D153V mutations upon viral growth of H3N8 influenza virus are associated with severe pathogenesis in human infections
Source: Influenza Other Respir Viruses. 2023 Jun 28;17(6):e13169. doi: 10.1111/irv.13169 (PMC10303683; doi:10.1111/irv.13169)
Supplement: Supplementary file 2 — Table S1. Nucleotide sequences of viruses closely related to A/Henan/4‐10/2022(H3N8) in the GenBank and GISAID databases. [file IRV-17-e13169-s003.docx]

**Table S1**: Nucleotide sequences of viruses closely related to A/Henan/4-10/2022(H3N8) in the GenBank and GISAID databases.

| **Gene** | **Virus** | **GenBank/GISAID Accession No.** | **Subtype** | **Identity (%)** |
| --- | --- | --- | --- | --- |
| HA | A/chicken/Hong Kong/21-17040/2021 | EPI2200708 or ON909097.1 | H3N8 | 98.82 |
|  | A/chicken/China/Guangdong_01/2022 | ON626399.1 | H3N8 | 98.64 |
|  | A/Changsha/1000/2022 (*human*) | EPI2035832 | H3N8 | 98.58 |
|  | A/duck/Guangdong/F352/2018 | EPI1328406 | H3N2 | 96.03 |
|  | A/duck/China/322D22/2018 | MN443576.1 or EPI1930531 | H3N2 | 95.86 |
| NA | A/chicken/China/Guangdong_01/2022 | ON626401.1 | H3N8 | 98.80 |
|  | A/chicken/Hong Kong/21-17040/2021 | EPI2200710 or ON909099.1 | H3N8 | 98.80 |
|  | A/Changsha/1000/2022 (*human*) | EPI2035833 | H3N8 | 98.60 |
|  | A/common teal/Sakhalin/66c/2020 | EPI1847526 | H3N8 | 97.14 |
|  | A/northern pintail/Alaska/870/2014 | EPI729736 | H3N8 | 97.07 |
|  | A/chicken/Anhui/FD3/2022 | EPI2047347 | H3N8 | 99.87 |
|  | A/chicken/Jiangsu/B314/2022 | EPI2047622 | H3N8 | 99.83 |
| PB2 | A/chicken/Jiangsu/A3123/2022 | EPI2047606 | H3N8 | 99.83 |
|  | A/chicken/Anhui/F3123/2022 | EPI2047331 | H3N8 | 99.83 |
|  | A/chicken/Jiangsu/C312/2022 | EPI2047642 | H3N8 | 99.79 |
|  | A/chicken/Jiangsu/B314/2022 | EPI2047623 | H3N8 | 99.96 |
|  | A/chicken/Jiangsu/A3123/2022 | EPI2047607 | H3N8 | 99.96 |
| PB1 | A/chicken/Guangdong/F0314/2022 | EPI2047567 | H3N8 | 99.96 |
|  | A/chicken/Jiangsu/C312/2022 | EPI2047643 | H3N8 | 99.91 |
|  | A/chicken/Anhui/FD3/2022 | EPI2047348 | H3N8 | 99.91 |
|  | A/chicken/Anhui/FD3/2022 | EPI2047349 | H3N8 | 100.00 |
|  | A/chicken/Jiangsu/C312/2022 | EPI2047644 | H3N8 | 99.95 |
| PA | A/chicken/Jiangsu/B314/2022 | EPI2047624 | H3N8 | 99.95 |
|  | A/chicken/Guangdong/F0314/2022 | EPI2047568 | H3N8 | 99.95 |
|  | A/chicken/Anhui/FE12/2022 | EPI2047365 | H3N8 | 99.95 |
|  | A/chicken/Anhui/FE12/2022 | EPI2047367 | H3N8 | 99.94 |
|  | A/chicken/Anhui/FD3/2022 | EPI2047352 | H3N8 | 99.94 |
| NP | A/chicken/Jiangsu/B314/2022 | EPI2047626 | H3N8 | 99.87 |
|  | A/chicken/Jiangsu/A3123/2022 | EPI2047610 | H3N8 | 99.87 |
|  | A/chicken/Guangdong/F0314/2022 | EPI2047573 | H3N8 | 99.87 |
| M | A/chicken/Anhui/FD3/2022 | EPI2047356 | H3N8 | 100.00 |
|  | A/chicken/Jiangsu/C312/2022 | EPI2047648 | H3N8 | 99.90 |
| M | A/chicken/Jiangsu/B314/2022 | EPI2047636 | H3N8 | 99.90 |
|  | A/chicken/Jiangsu/A3123/2022 | EPI2047612 | H3N8 | 99.90 |
|  | A/chicken/Anhui/FE12/2022 | EPI2047369 | H3N8 | 99.90 |
|  | A/chicken/Jiangsu/C312/2022 | EPI2047650 | H3N8 | 100.00 |
|  | A/chicken/Jiangsu/B314/2022 | EPI2047638 | H3N8 | 100.00 |
| NS | A/chicken/Guangdong/F0314/2022 | EPI2047579 | H3N8 | 100.00 |
|  | A/chicken/Anhui/FD3/2022 | EPI2047357 | H3N8 | 100.00 |
|  | A/chicken/Jiangsu/A3123/2022 | EPI2047613 | H3N8 | 99.89 |
