# Supplementary material for: Evidence that HA‐G228S and PB2‐D153V mutations upon viral growth of H3N8 influenza virus are associated with severe pathogenesis in human infections
Source: Influenza Other Respir Viruses. 2023 Jun 28;17(6):e13169. doi: 10.1111/irv.13169 (PMC10303683; doi:10.1111/irv.13169)
Supplement: Supplementary file 3 — Table S2. Molecular comparison of all viral residues of the two human H3N8 viruses. [file IRV-17-e13169-s002.docx]

**Table S2**. Molecular comparison of all viral residues of the two human H3N8 viruses.

| **Protein** | **Residue number†** | **A/Henan/4-10/2022‡** | **A/Henan/4-14/2022‡** | **A/Changsha/1000/2022** |
| --- | --- | --- | --- | --- |
| PB2 | 61 | K | K | R |
|  | 118 | F | F | Y |
|  | **153** | **D/V** | **D/V** | D |
|  | 299 | K | K | R |
|  | 340 | R | R | K |
|  | 355 | K | K | R |
|  | 409 | S | S | C |
|  | 473 | M | M | V |
|  | 570 | M | M | I |
|  | 627 | K | K | V |
|  | 676 | V | V | M |
|  | 702 | R | R | K |
| PB1 | 14 | V | V | A |
|  | 52 | K | K | N |
|  | 171 | M | M | I |
|  | 172 | N | N | D |
|  | 339 | V | V | I |
|  | 363 | K | K | R |
|  | 374 | E | E | A |
|  | 375 | S | S | N |
|  | 384 | A | A | S |
|  | 642 | N | N | S |
|  | 667 | I | I | V |
|  | 738 | K | K | E |
|  | 757 | K | K | N |
| PB1-F2 | 4 | Y | Y | C |
|  | 10 | R | R | Q |
|  | 21 | K | K | R |
|  | 37 | R | R | H |
|  | 41 | R | R | L |
|  | 45 | F | F | S |
| PA | 337 | T | T | A |
|  | 343 | S | S | A |
|  | 387 | V | V | I |
|  | 465 | I | I | V |
| HA | 25 | I | I | V |
|  | **228** | **G/S §** | **G/S §** | G |
|  | 269 | R | R | K |
|  | 278 | I | I | V |
|  | 335 | I | K | I |
|  | 384 | V | V | L |
|  | 489 | D | D | N |
|  | 525 | L | L | F |
| NP | 136 | L | L | M |
|  | 194 | I | I | V |
|  | 373 | A | A | T |
|  | 408 | V | V | I |
| NA | 6 | K | K | R |
|  | 12 | S | S | T |
|  | 17 | S | S | L |
|  | 27 | A | A | V |
|  | 37 | L | L | P |
|  | 42 | S | S | N |
|  | 46 | K | K | N |
|  | 78 | E | E | G |
|  | 83 | M | M | I |
|  | 191 | I | I | V |
| M1 | 46 | I | I | L |
| M2 | 10 | L | L | P |
|  | 12 | K | K | R |
|  | 13 | T | T | I |
|  | 20 | S | S | N |
|  | 28 | V | V | I |
|  | 42 | M | M | I |
|  | 89 | S | S | G |
| NS1  NS1 | length | 237 aa | 237 aa | 217 aa (218-237 truncated) |
|  | 44 | R | R | K |
|  | 72 | D | D | E |
|  | 73 | T | T | S |
|  | 76 | T | T | A |
|  | 80 | S | S | N |
|  | 127 | T | T | D |
|  | 205 | S | S | R |
|  | 212 | P | P | S |
|  | 218 | E | E | stop codon |
| NS2 | 47 | E | E | D |
|  | 60 | R | R | S |

^†^H3 numbering.

^‡^These viruses were isolated from the same patient (Case 1) on 10 and 14 April, 2022.

^§^G228S genomes are 37% in A/Henan/4-10/2022 and 64% in A/Henan/4-14/2022.

aa: amino acids. Bold characters: two mutations upon viral replication.
